# Review of the Literature on Ocular Complications Associated With Aromatase Inhibitor Use

**DOI:** 10.7759/cureus.17565

**Published:** 2021-08-30

**Authors:** Ibrahim Almafreji, Cameron Smith, Fraser Peck

**Affiliations:** 1 General Surgery, University Hospital Crosshouse, Kilmarnock, GBR; 2 Emergency Medicine, University Hospital Crosshouse, Kilmarnock, GBR; 3 Ophthalmology, Eastbourne District General Hospital, Eastbourne, GBR

**Keywords:** aromatase inhibitors, anastrozole, letrozole, exemestane, side-effects, maculopathy, retinopathy, ocular complications, ocular surface disease, meibomian gland dysfunction

## Abstract

Aromatase inhibitors (AIs), such as anastrozole, letrozole, and exemestane, are commonly used as adjuvant endocrine therapy in hormone-receptive breast cancer in postmenopausal women. Their adverse effects are well documented, except for visual disturbances. The purpose of this study was to review the current literature on ocular disease linked to AI use. Due to the scarcity of published data, any suggested ophthalmic adverse events were included to increase awareness of these drugs. The ocular side effects of tamoxifen use are well documented and were not included. Cases of rare side effects such as papilloedema, macular oedema, and uveitis associated with anastrozole and letrozole have been reported. Studies demonstrating retinopathy, in the form of crystalline retinopathy, hemicentral retinal artery occlusion, and retinal haemorrhages, are also noted. All three third-generation AIs can also lead to ocular surface diseases such as corneal epithelial changes, blepharitis, and keratitis. There is slightly more literature available regarding anastrozole-related ocular diseases. Although these are likely rare side effects, we recommend a high level of clinical suspicion when assessing patients with visual symptoms and on AIs. Larger prospective studies are necessary to further investigate these complications.

## Introduction and background

This review will discuss the effects on the eye of aromatase inhibitors (AIs). AIs and selective estrogen receptor modulators (SERMs), such as tamoxifen, are endocrine medications that are widely prescribed as adjuvant therapy for hormone receptor-positive breast cancer. SERMs act against breast cancer by occupying estrogen receptors while AIs inhibit the action of the enzyme aromatase, thereby suppressing estrogen levels in postmenopausal women [1].

Estrogenic activity directly impacts various physiological functions, notably visual function. Estrogen receptors are present within the anterior and posterior segments of the eye and in the lacrimal and meibomian glands responsible for protecting the surface of the eye [2,3]. As they are also present throughout the central nervous system, changes in estrogen levels could affect visual processing [4].

AIs can be broadly divided into two classes: non-steroidal and steroidal. Anastrozole and letrozole are third-generation non-steroidal AIs, while exemestane is of the steroidal class [1,5]. The adverse effects from their long-term use are well documented and include joint disorders, skeletal complications, and cardiotoxicity [6]. The side effect profile, including visual complications of tamoxifen, is well documented [1]. On the contrary, literature regarding such disturbances linked to AIs is scarce. Therefore, we set out to conduct a comprehensive literature review on the ophthalmic disorders related to AI use by searching PubMed and Embase databases using the aforementioned keywords.

## Review

Methodology

The literature search was conducted on three databases (Pubmed, Cochrane, Embase) using a combination of these keywords “Letrozole,” “Anastrozole,” “Exemestane,” “Aromatase inhibitors,” “Maculopathy,” “Retinopathy,” “Ophthalmic disorders,” and/or “Ocular disease.” Full-text articles published in any year were included. Due to the scarcity of published data, any suggested ophthalmic side effects were included to increase awareness of these drugs. Articles not available in English, duplicate articles, and those which did not present an ocular complication with the use of one of the AIs were excluded. A total of seven case reports, five randomized controlled trials (RCTs), and two observational studies were discussed in this review.

Results

A total of seven case reports, five RCTs, and two observational studies are discussed in this review. The results are summarised in Table 1.

**Table 1:** Results of the literature review. AI: aromatase inhibitor; RNFL: retinal nerve fibre length; CME: cystoid macular oedema

Author	No. of patients	AI used	Findings
Coppes et al. [7]	1	Anastrozole	Severe bilateral optic disc swelling and impaired visual acuity. Near-complete resolution of swelling after discontinuing anastrozole
Sathiamoorthi et al. [8]	1	Anastrozole	Bilateral CME and uveitis. Treated with difluprednate ophthalmic emulsion drops. The patient continued on anastrozole
Moschos et al. [9]	1	Letrozole	Unilateral macular edema. Treated with intravitreal injection of ranibizumab. Macular oedema improved
Moschos et al. [10]	41 breast cancer patients, 40 healthy control subjects	All AIs	AIs exhibited a significant decrease in RNFL thickness (average, superior and inferior), retinal response density, and visual acuity compared to healthy controls
Weider et al. [11]	1	Anastrozole	Bilateral parafoveal retinal crystalline deposits causing blurred vision. No change in vision after follow-up
Karagöz et al. [12]	1	Anastrozole	Sudden painless loss of vision. Hemicentral retinal artery occlusion
Eisner et al. [13]	35 anastrozole users, 53 control subjects	Anastrozole	Anastrozole use appears to be associated with an increased prevalence of retinal haemorrhages. Four anastrozole users (two flame and two blot haemorrhages)
Eisner et al. [14]	27 anastrozole users, 40 control subjects	Anastrozole	The foveas of women using anastrozole seem to be prone to more tractional force than the foveas of women not using AIs
Epstein [15]	2	Exemestane	Reduced visual acuity in two myopic patients who recently commenced exemestane
Papathanassiou et al. [16]	1	Exemestane	Bilateral corneal intraepithelial microcysts. One-year follow-up showed unchanged corneal features, and the visual acuity remained unaffected
Chatziralli et al. [17]	41 breast cancer patients, 40 healthy control subjects	All AIs	Patients receiving AIs presented blepharitis in 75% and meibomian gland dysfunction in 42.5%. Superficial punctate keratitis and conjunctival injection were also present in 30% and 22.5%
Ağın et al. [18]	13	All AIs	AI therapy causes deteriorations in several ocular surface parameters and corneal structural changes
Bicer et al. [19]	50 anastrozole and letrozole users, 50 healthy controls	Anastrozole, letrozole	Ocular Surface Disease Index scores were lower, and fluorescein breakup time measurements were higher in controls. Schirmer test scores were higher in controls than AI group
Turaka et al. [20]	41	All AIs	Patients receiving AIs presented blepharitis in 73%, poor tear function in 29%, conjunctival injection in 22%, and superficial keratitis in 29%

Discussion

The incidence of certain side effects, especially visual disturbance, related to AI use is quite variable [6]. Literature on ocular complications linked to anastrozole use was slightly more prevalent compared to other AIs. Coppes et al. first published a case of papilloedema and macular oedema secondary to the use of anastrozole [7]. This was possibly due to changes in estrogen levels leading to vasculature permeability and optic nerve swelling. In this case, the patient experienced near-complete resolution of swelling in the affected eye with a course of prednisolone and by stopping anastrozole. Sathiamoorthi et al. reported a case of uveitis and macular oedema in a patient on long-term anastrozole therapy. Systemic inflammatory processes and other drug causes were ruled out, making anastrozole the likely culprit in the case [8]. The therapy consisted of difluprednate eye drops and resulted in an almost complete resolution of symptoms. Letrozole, which is another commonly used AI, has also been reported to inflict macular oedema [9]. Ocular coherence tomography showed intraretinal fluid and a significant increase in retinal thickness in the foveal and parafoveal areas. The patient responded to stopping letrozole and intravitreal injection of ranibizumab.

Moschos et al. investigated the impact of AIs on macular, retinal, and optic nerve function. The study concluded that AIs significantly decrease retinal nerve fibre layer thickness, retinal response density, and visual acuity [10]. There has also been evidence of crystalline retinopathy in a patient who was six months into anastrozole treatment [11]. Her presenting complaint was blurred vision which did not change following six months of follow-up while continuing treatment. Moreover, a rare report of hemicentral retinal artery occlusion was diagnosed on fluorescein angiography in a patient presenting with sudden painless vision loss [12]. Anastrozole use has been linked to thromboembolic and vascular complications [6]. This is further supported by Eisner et al. who concluded there was an increased prevalence of retinal haemorrhages with this drug [13]. These haemorrhages appeared within the posterior pole, two of which were flame haemorrhages and the other two were blot haemorrhages. One possible explanation for this phenomenon would be systemic vascular compromise due to estrogen depletion [13]. Another possibility is vitreoretinal traction for which evidence has been illustrated by Eisner et al. [14]. Their results showed that the foveas of women using AIs were altered with signs of displacement. Because this study was cross-sectional and contained subjects with excellent acuity, the ability of the traction to cause acuity loss remains unknown. However, the authors of the study conjectured that the combination of myopia and tractional changes could reduce visual function. Epstein presented a case of visual deterioration in two Chinese patients with pre-existing myopia who had recently started exemestane [15]. As aromatase plays a role in maintaining retinal structure and function, this drop in acuity was likely due to this AI.

Exemestane has also been reported to cause ocular surface disease, specifically corneal epithelial changes [16]. Upon routine eye examination, bilateral intraepithelial microcysts were revealed. The patient’s visual acuity remained unaffected despite the persisting corneal changes. Chatziralli et al. investigated the correlation between AI use and ocular surface disease [17]. Slit-lamp examination revealed blepharitis and meibomian gland dysfunction in 75% of the AI users compared to 42.5% of the control group. Cases of superficial punctate keratitis and conjunctival injection were also present. A recent study by Agin et al. supported the deteriorations caused by AI on ocular surface parameters and corneal structural integrity [18]. There has also been evidence that AIs (anastrozole, letrozole) may negatively affect tear function even more so than tamoxifen [19]. This sentiment was echoed by Turaka et al. who also put forth evidence that AI may cause blepharitis, poor tear function, conjunctival injection, and superficial punctate keratitis [20]. Overall, 9.5% of the patients in this study exhibited dry eye syndrome.

## Conclusions

Although visual disturbance is a possible side effect of AI use, its incidence is quite variable. The literature demonstrates evidence of retinopathy and maculopathy secondary to anastrozole and letrozole. Management includes stopping the causative medication. All three AIs have been reported to cause ocular surface disease.

Overall, ocular complications secondary to AI use are documented but are reported less commonly compared to tamoxifen which is known to have a worse side effect profile. Prompt ophthalmoscopic evaluation and a high index of suspicion are recommended when assessing a patient symptomatic of dry eye or visual disturbance receiving AIs. Case reports, cross-sectional, and observational studies comprised the articles included in this review. Larger prospective studies are certainly needed to analyse the severity of ocular complications and compare the incidence between each AI.
